# Motorcycle-Related Mortality in Brazil, 2000-2023: National Trends by Region, Sex, and Age

**DOI:** 10.7759/cureus.115172

**Published:** 2026-08-25

**Authors:** Jose Ilton Silva, Marcio Paulo C Salgueiro, Jessica N Tsurumaki, Giulia Maria T Siqueira, Ghiordana Milena D Lopes Guimarães, Ricardo Antonio P Mendes, Eduarda S Guedes, João B Braga Neto, Larissa B Ferreira, Thiago C Barreto

**Affiliations:** 1 Faculty of Medicine, Universidade Federal Rural do Semi-Árido, Mossoró, BRA; 2 Faculty of Medicine, Centro Universitário São Camilo, São Paulo, BRA; 3 Medicine, Universidade Nove de Julho, São Paulo, BRA; 4 Medical School, Universidade Fundação Oswaldo Aranha, Rio de Janeiro, BRA; 5 Faculty of Medicine, Universidade de Rio Verde, Goiânia, BRA; 6 Faculty of Medicine, Universidade Católica de Brasília, Brasília, BRA; 7 Faculty of Medicine, Universidade do Grande Rio (Unigranrio), Rio de Janeiro, BRA; 8 Faculty of Medicine, Faculdade de Ciências Médicas da Paraíba, João Pessoa, BRA; 9 Faculty of Medicine, Universidade Federal do Tocantins, Palmas, BRA; 10 Medical School, Federal University of Goiás, Goiânia, BRA

**Keywords:** age and sex differences, brazil, mortality trends, motorcycle accidents, road traffic injuries, time-series analysis

## Abstract

Background

Motorcyclist mortality has increased substantially in Brazil, but its long-term trajectory and variation across population groups remain incompletely characterized. We examined national trends from 2000 through 2023 by geographic region, sex, and age group.

Methods

We conducted a nationwide ecological time-series study of deaths among Brazilian residents assigned to International Classification of Diseases, 10th Revision (ICD-10) codes V20-V29 in the Mortality Information System. Population denominators were obtained from the Brazilian Institute of Geography and Statistics. Crude and directly age-standardized rates were calculated using the 2022 census population as the standard. Weighted segmented log-linear regression estimated annual percentage changes (APCs) and average annual percentage changes with 95% CIs. Joinpoints were selected using a BIC-type criterion; 2020 was not prespecified. Differences in average slopes were assessed using negative binomial models with population offsets and year-by-group interactions.

Results

Between 2000 and 2023, 225,530 motorcyclist deaths were recorded. The age-standardized mortality rate increased from 1.40 to 6.25 per 100,000 population. Mortality increased during 2000-2006 (APC, 16.7%; 95% CI, 16.0%-17.3%), 2006-2010 (9.7%; 9.1%-10.2%), and 2010-2014 (2.9%; 2.5%-3.4%), declined during 2014-2020 (−2.3%; −2.6% to −2.0%), and increased during the 2020-2023 segment (4.9%; 4.2%-5.6%). In 2023, rates were highest in the Northeast (9.47), Central-West (8.80), and North (8.45). Men accounted for 89.2% of deaths. Adults aged 20-24 years had the highest 2023 rate and the largest absolute increase, whereas adults aged 50 years or older experienced the largest relative increases from lower baseline rates. Average log-linear slopes differed by region (Holm-adjusted p=0.0016) and age group (Holm-adjusted p<0.001). The year-by-sex interaction did not provide evidence of a difference in the average slope (Holm-adjusted p=0.30), although the segmented trajectories differed in timing.

Conclusions

Motorcyclist mortality increased more than fourfold, with nonlinear temporal changes and marked regional and age-related heterogeneity. Young adults bore the greatest absolute burden, whereas relative increases were largest at older ages from lower baseline rates. Population-based rates cannot distinguish changes in motorcycle exposure from changes in exposure-adjusted risk.

## Introduction

Road traffic injuries remain a major cause of preventable death and disability, with a disproportionate burden in low- and middle-income countries. In Brazil, reductions in overall road traffic mortality have not been shared equally across road users. Motorcyclists account for an increasing share of road traffic deaths, with a particularly high burden among men, young adults, and populations in the North, Northeast, and Central-West regions [1-3]. Because motorcycles are widely used for personal mobility and income-generating activities, motorcyclist mortality has important implications for public health, transport policy, and the working-age population.

National studies documented a marked increase in motorcyclist mortality during the 1990s and 2000s. From 1996 to 2009, the age-standardized mortality rate among motorcyclists increased approximately eightfold, with the largest increases in less affluent regions [2]. A separate national study reported an increase of more than 300% in mortality among motorcycle occupants between 2000 and 2008, while mortality among several other road-user groups declined or increased less sharply [3]. Together, these findings established motorcyclists as an increasingly important component of Brazil’s road traffic mortality burden.

More recent evidence suggests that this trajectory has not been uniform. Municipal-level analyses through 2018 identified persistent high-mortality clusters in the Northeast, North, and Central-West despite declines in overall road traffic mortality, whereas estimates for tropical Latin America suggested decreasing motorcyclist mortality after the early 2010s [4,5]. State- and city-level studies have reported continued increases, stabilization, or declines, indicating that national averages may obscure substantial geographic and demographic heterogeneity [6-9]. Mortality among female motorcyclists has also increased in many Brazilian municipalities, although men continue to account for most deaths [10]. However, previous studies have not provided a national analysis extending through 2023 that jointly examines long-term trends across geographic regions, sexes, and age groups.

We therefore examined motorcyclist mortality in Brazil from 2000 to 2023 using national vital registration data. The primary objectives were to characterize long-term changes in crude and age-standardized mortality rates, identify inflection points in the national trajectory, and determine whether trends differed across geographic regions, sexes, and age groups. 

## Materials and methods

Study design and data sources

We conducted a nationwide ecological time-series study of motorcyclist mortality in Brazil from 2000 through 2023. Mortality data were obtained from the Brazilian Mortality Information System (Sistema de Informações sobre Mortalidade, SIM), maintained by the Brazilian Ministry of Health and accessed through the DATASUS TabNet platform on July 14, 2026 [11].

We included deaths of Brazilian residents occurring between January 1, 2000, and December 31, 2023, for which the underlying cause of death was classified under International Classification of Diseases, 10th Revision (ICD-10) codes V20-V29, encompassing motorcycle drivers and passengers injured in transport crashes. Under the ICD-10 transport classification, V20-V29 encompasses riders of motorcycles and related motorized two-wheel vehicles, including mopeds, motor scooters, and motorized bicycles. The mortality database does not provide sufficient vehicle-level detail to consistently distinguish conventional motorcycles from specific electrically powered two-wheel vehicle subtypes across the full study period [12].

Annual population denominators were obtained from the Brazilian Institute of Geography and Statistics (IBGE) Population Projections, 2024 Revision, accessed on July 5, 2026 [13]. This revised demographic series provides annual population estimates by sex and age throughout the study period; no interpolation between census years was performed by the investigators. Mortality and population data were linked by calendar year, sex, age group, and geographic region. Deaths were assigned to geographic regions according to the place of residence of the deceased.

Study variables

The primary outcome was the annual age-standardized mortality rate among motorcycle riders per 100,000 population. Secondary outcomes included the annual number of deaths, crude mortality rates, and mortality rates according to sex, age group, and geographic region.

Brazil was divided into its five official geographic regions: North, Northeast, Central-West, Southeast, and South. Age-specific analyses were conducted using the following groups: 15-19 years, 20-24 years, 25-29 years, 30-39 years, 40-49 years, 50-59 years, and 60 years or older. Individuals younger than 15 years were excluded from the age-specific trend analyses because of the small number of deaths in this population.

Records with missing information on sex were included in the overall analysis but excluded from sex-specific analyses. Records with missing age were included in crude mortality estimates but excluded from age-specific and age-standardized analyses.

Mortality rates

Crude mortality rates were calculated by dividing the annual number of deaths by the corresponding resident population and multiplying by 100,000.

Age-standardized mortality rates were calculated by the direct method using the age distribution of the Brazilian population reported in the 2022 national census as the standard population [14]. Five-year age groups were used for standardization.

Ninety-five percent confidence intervals for crude rates were calculated assuming a Poisson distribution. Confidence intervals for directly age-standardized rates were calculated using the gamma method of Fay and Feuer [15].

Temporal trend analysis

Temporal trends were modeled using weighted segmented log-linear regression applied to annual age-standardized mortality rates. Models were weighted by the inverse variance of each annual rate. For overall age-standardized rates, variances were estimated using the Dobson method; Poisson-based variances were used for age-specific rates.

The number and location of joinpoints were determined through a sequential search minimizing a Bayesian information criterion-type measure, with a maximum of four joinpoints and a minimum segment length of three years. The year 2020 was not prespecified as a breakpoint.

Annual percentage changes (APCs) were estimated for each segment, and average annual percentage changes (AAPCs) were calculated over the full 2000-2023 period as duration-weighted averages of the segment-specific changes [16]. Estimates are presented with 95% confidence intervals. Trends were classified as increasing when the APC was greater than zero and the 95% confidence interval excluded zero, decreasing when the APC was below zero and the 95% confidence interval excluded zero, and stable when the confidence interval included zero.

Comparison of trends between population groups

Differences in mortality trends across geographical regions, sexes, and age groups were assessed using generalized linear models for annual death counts. Calendar year, population group, and a year-by-group interaction term were included in each model, with the logarithm of the corresponding population used as an offset.

Poisson regression was initially fitted. Model dispersion was assessed using the residual deviance and Pearson chi-square statistic. When overdispersion was present, negative binomial regression was used.

The statistical significance of each year-by-group interaction was evaluated using a likelihood-ratio chi-square test comparing models with and without the interaction term. Results were reported as annual percentage changes or rate ratios with 95% confidence intervals.

Sensitivity analyses

Robustness of the main findings was assessed using several alternative analytical specifications. Temporal trends were re-estimated using crude rather than age-standardized mortality rates. For subgroup analyses, Poisson and negative binomial models were compared, with negative binomial regression used when overdispersion was present. The long-term national trend was also re-estimated after excluding observations from 2020 and 2021 to assess the influence of these years on the overall trajectory.

Residual serial autocorrelation was evaluated in the national time series. When present, Prais-Winsten regression of log-transformed annual mortality rates was used as a sensitivity analysis. The stability of the principal inflection points was additionally examined by varying the maximum number of permitted joinpoints.

Statistical analysis

All data management, statistical analyses, and figures were performed in R, version 4.6.1 (R Foundation for Statistical Computing, Vienna, Austria). Generalized linear models were fitted using the base stats package, and negative binomial models using the MASS package, version 7.3-66. Differences in average temporal slopes across geographic regions, sexes, and age groups were evaluated using likelihood-ratio chi-square tests comparing models with and without year-by-group interaction terms. Because three prespecified global interaction tests were performed, corresponding p values were adjusted for multiple comparisons using the Holm-Bonferroni method.

All statistical tests were two-sided, and estimates are reported with 95% confidence intervals. A two-sided p value <0.05 was considered statistically significant.

Ethical considerations

The study used publicly available, aggregated, and anonymized secondary data. No individually identifiable information was accessed. Under Resolution 510/2016 of the Brazilian National Health Council, studies conducted exclusively with public-domain aggregate data do not require review by a research ethics committee.

## Results

Overall mortality burden

Between 2000 and 2023, 225,530 motorcyclist deaths were recorded in Brazil. Age was available for 225,229 deaths (99.87%), and sex was available for 225,510 (99.99%). Among records with known sex, 201,225 deaths (89.2%) occurred in men and 24,285 (10.8%) in women.

The annual number of deaths increased from 2,465 in 2000 to 13,477 in 2023, representing a 5.5-fold increase. Over the same period, the crude mortality rate increased from 1.45 to 6.23 deaths per 100,000 population. After direct age standardization to the 2022 Brazilian census population, the mortality rate increased from 1.40 deaths per 100,000 population (95% CI, 1.34-1.46) in 2000 to 6.25 (95% CI, 6.14-6.35) in 2023, corresponding to a 4.5-fold increase (Table 1).

**Table 1:** Deaths and mortality rates among motorcycle riders, Brazil and geographical regions, 2000 and 2023 *Rates per 100 000 population. Age-standardized rates were directly standardized to the age distribution of the 2022 Brazilian census population; 95% CI calculated using the gamma method of Fay and Feuer [15].

Area	Deaths, 2000	Crude rate, 2000*	Age-standardised rate, 2000* (95% CI)	Deaths, 2023	Crude rate, 2023*	Age-standardised rate, 2023* (95% CI)
Brazil - Overall	2465	1.45	1.4 (1.34-1.46)	13477	6.23	6.25 (6.14-6.35)
Brazil - Men	2250	2.69	2.61 (2.5-2.73)	11864	11.23	11.23 (11.02-11.43)
Brazil - Women	215	0.25	0.24 (0.2-0.27)	1613	1.46	1.47 (1.4-1.54)
North	255	1.96	2.1 (1.83-2.42)	1529	8.38	8.45 (8.03-8.9)
Northeast	806	1.68	1.71 (1.59-1.84)	5366	9.54	9.47 (9.22-9.73)
Southeast	579	0.79	0.72 (0.66-0.78)	3313	3.79	3.78 (3.65-3.91)
South	503	2.01	1.86 (1.7-2.04)	1765	5.78	5.77 (5.51-6.05)
Central-West	322	2.77	2.62 (2.32-2.96)	1504	8.8	8.8 (8.36-9.26)

National temporal trends

**Figure 1:**
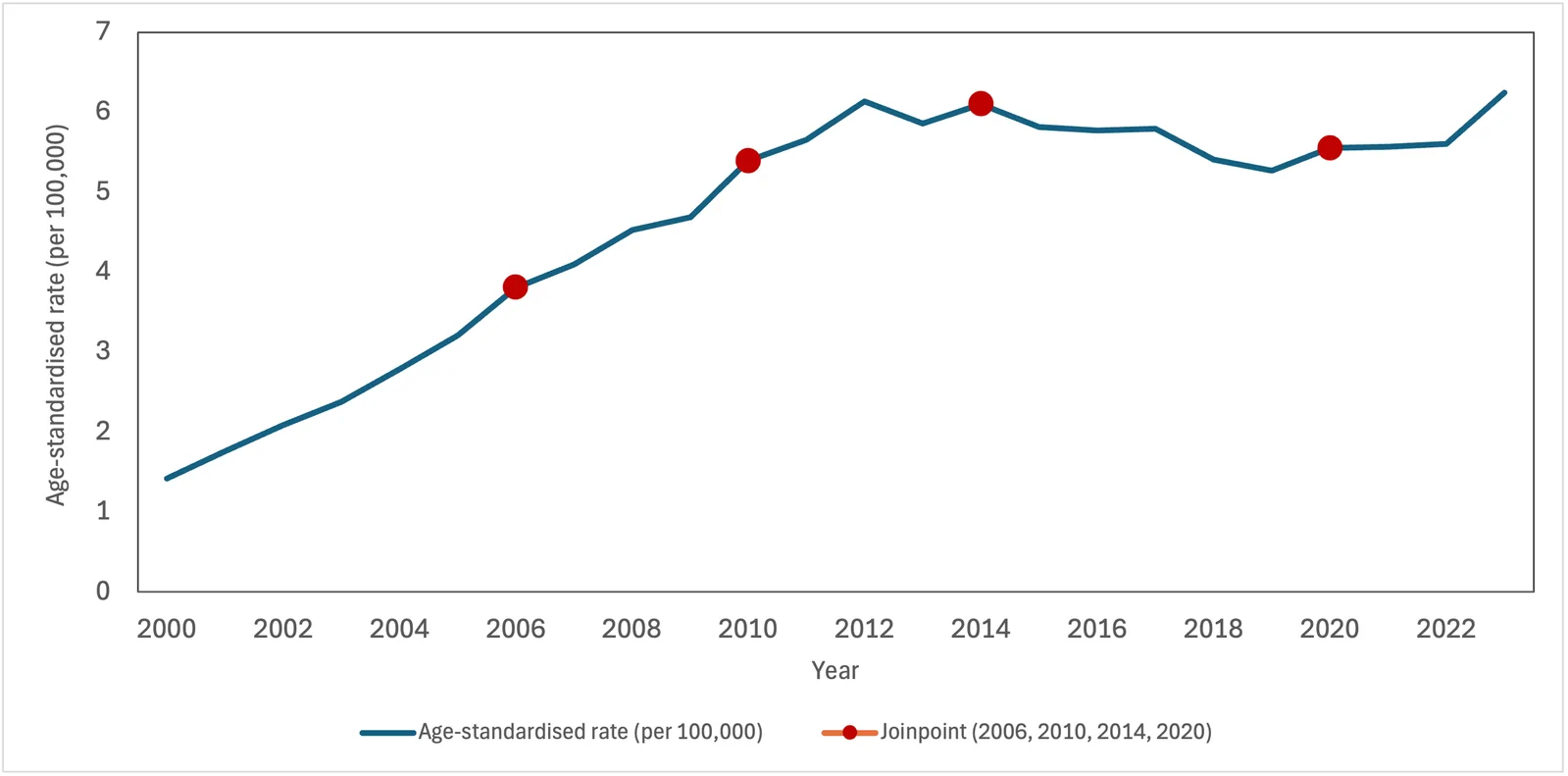
National age-standardized mortality rate, 2000-2023, with joinpoint segments marked

The national trajectory was nonlinear. Segmented log-linear analysis identified changes in trend in 2006, 2010, 2014, and 2020 (Figure 1). The age-standardized mortality rate increased by 16.7% per year (95% CI, 16.0%-17.3%) between 2000 and 2006, by 9.7% per year (95% CI, 9.1%-10.2%) between 2006 and 2010, and by 2.9% per year (95% CI, 2.5%-3.4%) between 2010 and 2014. The decline during 2014-2020 (APC, −2.3% per year; 95% CI, −2.6% to −2.0%) was followed by an increasing 2020-2023 segment (APC, 4.9% per year; 95% CI, 4.2%-5.6%). The AAPC over the complete study period was 6.3% per year (95% CI, 6.1%-6.6%). The age-standardized mortality rate reached 6.25 deaths per 100,000 population in 2023, the highest value observed during the study period (Table 2).

**Table 2:** Joinpoint analysis of age-standardized mortality rates among motorcycle riders, Brazil and geographical regions, 2000-2023 APC indicates annual percentage change within each joinpoint-defined segment; AAPC, average annual percentage change across 2000-2023, weighted by the duration of each segment; CI, confidence interval. APCs and AAPCs were estimated using segmented log-linear regression. Joinpoints were selected through sequential search minimizing a BIC-type criterion, with a maximum of four joinpoints and a minimum segment length of three years. AAPCs were calculated as duration-weighted averages of segment-specific changes.

Area / Sex	Period	APC (%)	APC 95% CI	Trend	AAPC 2000-2023	AAPC 2000-2023 (95% CI)
Brazil - Overall	2000-2006	16.7%	16.0% to 17.3%	Increasing	6.3%/year	6.1% to 6.6%
	2006-2010	9.7%	9.1% to 10.2%	Increasing		
	2010-2014	2.9%	2.5% to 3.4%	Increasing		
	2014-2020	-2.3%	-2.6% to -2.0%	Decreasing		
	2020-2023	4.9%	4.2% to 5.6%	Increasing		
Brazil - Men	2000-2005	17.5%	16.7% to 18.3%	Increasing	6.1%/year	5.9% to 6.4%
	2005-2010	9.9%	9.4% to 10.5%	Increasing		
	2010-2013	4.5%	3.8% to 5.2%	Increasing		
	2013-2019	-2.6%	-2.9% to -2.3%	Decreasing		
	2019-2023	2.8%	2.3% to 3.4%	Increasing		
Brazil - Women	2000-2003	27.1%	21.7% to 32.7%	Increasing	8.3%/year	7.5% to 9.2%
	2003-2010	13.2%	12.0% to 14.4%	Increasing		
	2010-2013	3.2%	1.5% to 5.1%	Increasing		
	2013-2020	-0.7%	-1.5% to 0.0%	Stable		
	2020-2023	7.3%	5.3% to 9.3%	Increasing		
North	2000-2013	8.9%	8.3% to 9.5%	Increasing	5.4%/year	4.8% to 6.0%
	2013-2017	2.2%	0.8% to 3.6%	Increasing		
	2017-2020	-6.3%	-8.2% to -4.4%	Decreasing		
	2020-2023	7.1%	4.8% to 9.5%	Increasing		
Northeast	2000-2005	20.7%	19.3% to 22.1%	Increasing	4.2%/year	3.8% to 4.6%
	2005-2011	-1.2%	-1.8% to -0.7%	Decreasing		
	2011-2017	1.2%	0.8% to 1.7%	Increasing		
	2017-2020	-5.4%	-6.3% to -4.5%	Decreasing		
	2020-2023	6.1%	4.9% to 7.3%	Increasing		
Southeast	2000-2008	21.0%	20.2% to 21.8%	Increasing	6.9%/year	6.5% to 7.3%
	2008-2011	1.8%	0.5% to 3.0%	Increasing		
	2011-2014	-1.6%	-2.8% to -0.4%	Decreasing		
	2014-2019	-3.4%	-4.0% to -2.7%	Decreasing		
	2019-2023	4.4%	3.3% to 5.4%	Increasing		
South	2000-2008	12.7%	11.8% to 13.6%	Increasing	4.4%/year	3.8% to 4.9%
	2008-2012	3.0%	1.8% to 4.3%	Increasing		
	2012-2015	-6.4%	-8.0% to -4.8%	Decreasing		
	2015-2019	0.6%	-0.7% to 1.9%	Stable		
	2019-2023	2.1%	0.8% to 3.5%	Increasing		
Central-West	2000-2005	16.1%	13.8% to 18.5%	Increasing	5.4%/year	4.7% to 6.1%
	2005-2009	10.1%	8.3% to 11.9%	Increasing		
	2009-2014	1.3%	0.2% to 2.3%	Increasing		
	2014-2020	-1.9%	-2.7% to -1.1%	Decreasing		
	2020-2023	4.2%	2.2% to 6.3%	Increasing		

Regional patterns

**Figure 2:**
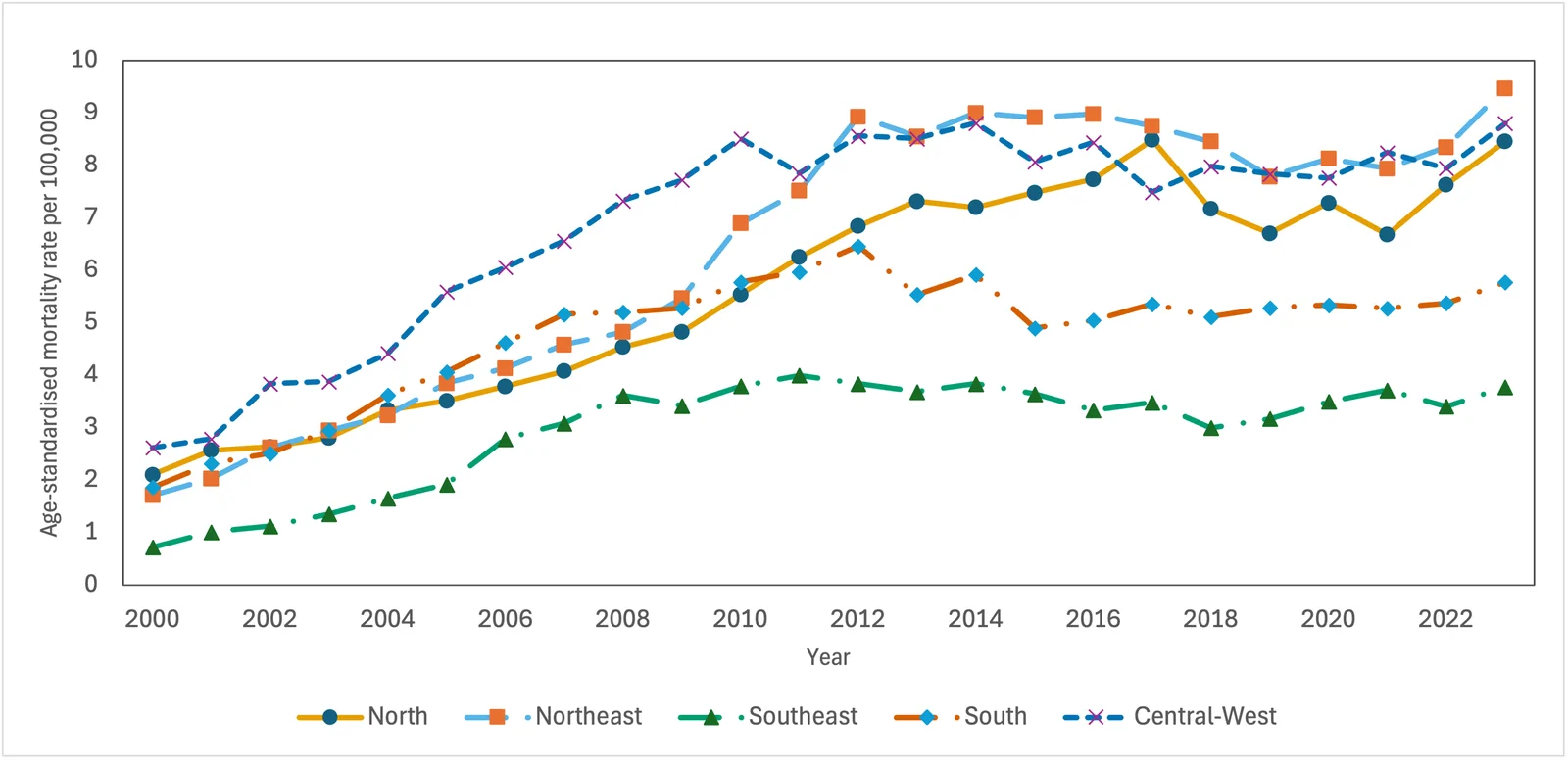
Age-standardised mortality rate by region, 2000-2023

Mortality increased substantially in all five geographic regions, although the magnitude and timing of the changes differed (Figure 2). In 2023, the Northeast had the highest age-standardized mortality rate, at 9.47 deaths per 100,000 population (95% CI, 9.22-9.73), followed by the Central-West at 8.80 (95% CI, 8.36-9.26), the North at 8.45 (95% CI, 8.03-8.90), the South at 5.77 (95% CI, 5.51-6.05), and the Southeast at 3.78 (95% CI, 3.65-3.91).

All regions showed an initial period of increasing mortality, followed by attenuation or decline and a subsequent increase beginning in 2019 or 2020. The average annual percentage change ranged from 4.2% per year in the Northeast to 6.9% per year in the Southeast. The corresponding estimates were 5.4% per year in both the North and Central-West and 4.4% per year in the South (Table 2).

Average log-linear slopes differed across geographic regions (likelihood-ratio χ²(4)=19.2; p=0.0008; Holm-adjusted p=0.0016).

Sex-specific trends

**Figure 3:**
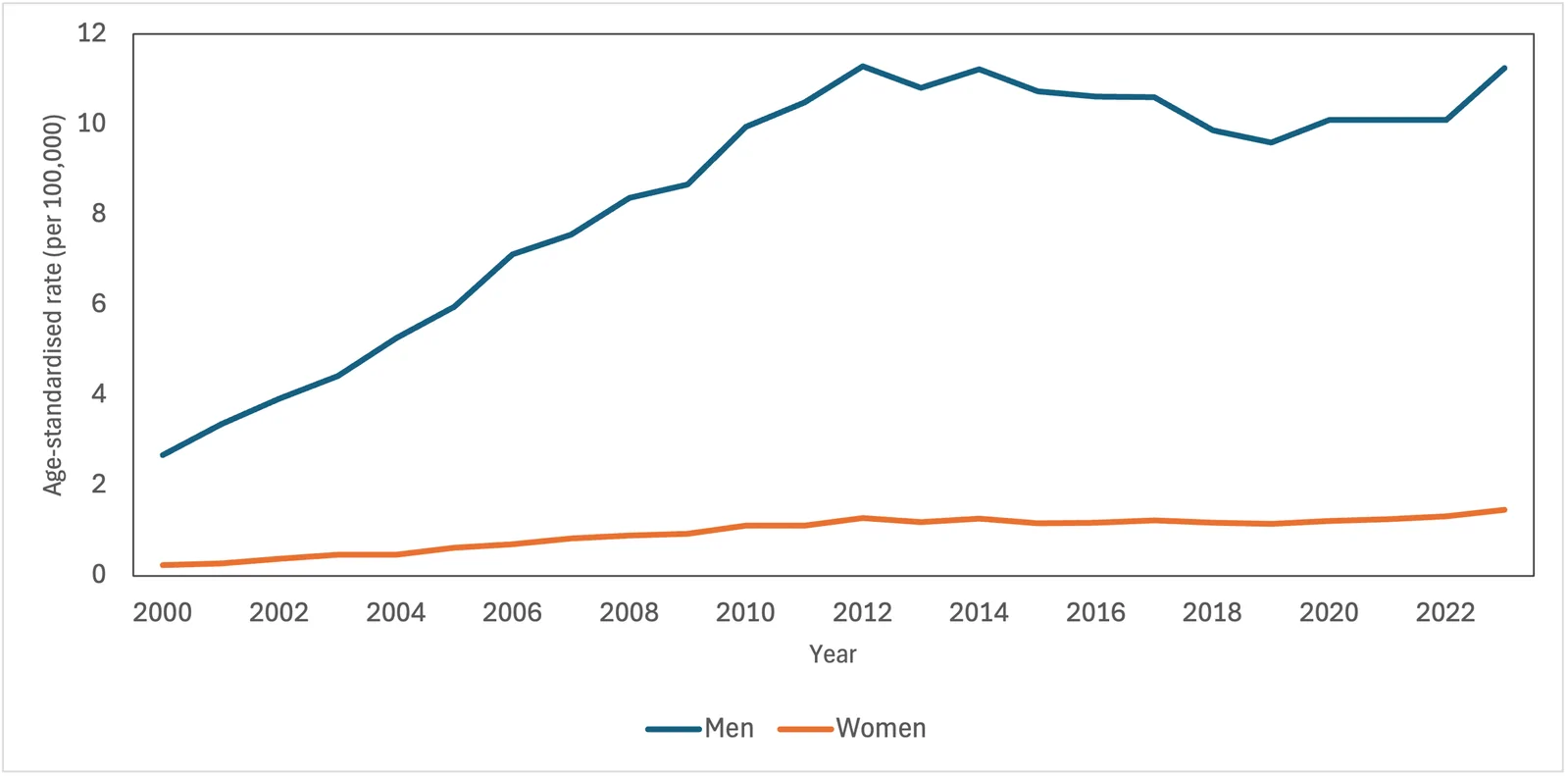
Age-standardised mortality rate by sex, Brazil, 2000-2023

Mortality rates remained substantially higher among men throughout the study period. The age-standardized mortality rate among men increased from 2.61 deaths per 100,000 population (95% CI, 2.50-2.73) in 2000 to 11.23 (95% CI, 11.02-11.43) in 2023. Among women, the corresponding rate increased from 0.24 (95% CI, 0.20-0.27) to 1.47 (95% CI, 1.40-1.54) (Table 1 and Figure 3).

The male-to-female rate ratio decreased from approximately 11.0 in 2000 to 7.6 in 2023. The average annual percentage change was numerically greater among women than among men, at 8.3% and 6.1% per year, respectively. The year-by-sex interaction did not provide evidence of a difference in the average log-linear slope over the full study period (likelihood-ratio χ²(1)=1.06; p=0.3039; Holm-adjusted p=0.3039).

Both sexes showed rapid increases during the early study period, followed by attenuation or decline during the 2010s and renewed growth in the most recent segment. From 2020 to 2023, mortality increased by 7.3% per year (95% CI, 5.3-9.3) among women and by 2.8% per year (95% CI, 2.3-3.4) among men (Table 2).

Age-specific trends

Age-specific temporal patterns differed substantially across age groups (Table 3). Adults aged 20-24 years had the highest mortality rate in 2023, at 13.79 deaths per 100,000 population, and the largest absolute increase over the study period, from 3.93 in 2000 to 13.79 in 2023, corresponding to an increase of 9.87 deaths per 100,000 population. Adults aged 25-29 years had the second highest 2023 rate, at 10.86 per 100,000 population.

**Table 3:** Age-specific temporal trends and changes in motorcyclist mortality rates in Brazil, 2000–2023 Rates are expressed as deaths per 100,000 population. APC indicates annual percentage change within the most recent segment identified by weighted segmented log-linear regression; AAPC, average annual percentage change over the entire 2000–2023 study period; CI, confidence interval. Absolute change was calculated as the 2023 rate minus the 2000 rate, and the rate ratio as the 2023 rate divided by the 2000 rate. AAPCs were calculated as duration-weighted averages of the segment-specific annual percentage changes. Trends were classified as increasing or decreasing when the 95% CI for the APC excluded zero and as stable when the 95% CI included zero.

Age group	AAPC 2000-2023	Most recent segment	APC in most recent segment (95% CI)	Trend (most recent segment)	Rate 2000*	Rate 2023*	Absolute change 2000→2023	Ratio 2023/2000	AAPC 2000-2023 (95% CI)
15–19 years	5.7%/year	2018-2023	-0.4% (-1.6 to 0.9)	Stable	2.03	7.33	5.30	3.6x	5.1% to 6.4%
20–24 years	5.2%/year	2019-2023	2.9% (1.8 to 4.0)	Increasing	3.93	13.79	9.87	3.5x	4.7% to 5.7%
25–29 years	5.0%/year	2020-2023	6.1% (4.2 to 8.0)	Increasing	3.26	10.86	7.60	3.3x	4.4% to 5.6%
30–39 years	5.6%/year	2020-2023	2.6% (1.1 to 4.0)	Increasing	2.23	8.50	6.27	3.8x	5.1% to 6.1%
40–49 years	7.8%/year	2020-2023	5.4% (3.7 to 7.1)	Increasing	1.33	7.68	6.35	5.8x	7.2% to 8.5%
50–59 years	9.6%/year	2020-2023	7.1% (5.0 to 9.2)	Increasing	0.85	6.60	5.75	7.8x	8.6% to 10.5%
≥60 years	10.0%/year	2020-2023	9.1% (6.5 to 11.7)	Increasing	0.37	3.50	3.13	9.4x	8.7% to 11.4%

**Figure 4:**
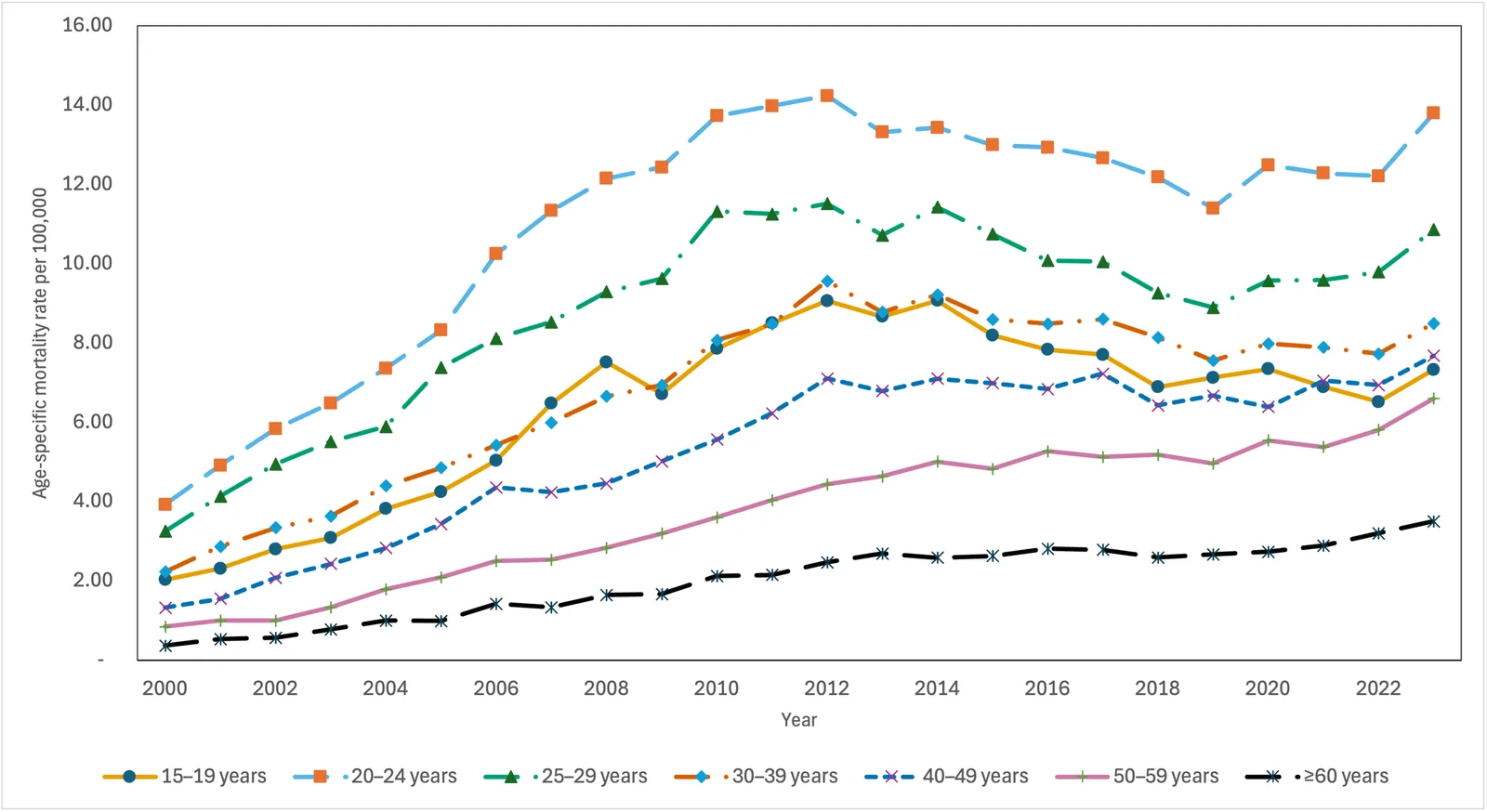
Age-specific motorcyclist mortality rates by age group, Brazil, 2000-2023

In contrast, the largest relative long-term increases occurred among older adults. The AAPC was 9.6% per year (95% CI, 8.6%-10.5%) among adults aged 50-59 years and 10.0% per year (95% CI, 8.7%-11.4%) among those aged 60 years or older. These increases occurred from substantially lower baseline rates: mortality increased from 0.85 to 6.60 per 100,000 population among adults aged 50-59 years and from 0.37 to 3.50 per 100,000 population among those aged 60 years or older (Figure 4).

In the most recent segments, mortality increased in all age groups except adolescents aged 15-19 years, among whom the trend was stable (APC, −0.4%; 95% CI, −1.6% to 0.9%). The steepest recent increases were observed among adults aged 60 years or older (APC, 9.1%; 95% CI, 6.5%-11.7%) and those aged 50-59 years (APC, 7.1%; 95% CI, 5.0%-9.2%).

The interaction between calendar year and age group was statistically significant (likelihood-ratio χ²(6)=44.3; p<0.0001; Holm-adjusted p<0.001), indicating differences in the average log-linear slopes across age groups.

Sensitivity analyses

The main findings were robust across alternative analytical specifications. Differences between crude and age-standardized estimates of the AAPC were smaller than 0.5 percentage points for Brazil and all five regions. Excluding 2020 and 2021 yielded an AAPC of 6.32% per year, compared with 6.33% when all years were included.

The conclusions of the subgroup interaction analyses were unchanged when quasi-Poisson models were used instead of negative binomial regression. Residual serial autocorrelation was present in the national single-slope model (Durbin-Watson statistic, 0.10). Prais-Winsten regression yielded an annual increase of 4.0% (95% CI, 3.7%-4.2%), consistent with the uncorrected estimate. The principal inflection years, particularly 2010, 2014, and 2020, remained stable when the maximum number of permitted joinpoints was varied.

The proportion of land-transport deaths assigned to V89 declined nationally from 36.8% in 2000 to 12.7% in 2023. Across regions, the proportion ranged from 20.7% to 47.8% in 2000 and from 4.0% to 19.6% in 2023 (Appendix).

## Discussion

Motorcyclist mortality in Brazil increased more than fourfold between 2000 and 2023, reaching its highest age-standardized rate in 2023. The national trajectory was nonlinear, with rapid growth through 2014, a decline until 2020, and renewed growth thereafter. Marked heterogeneity was observed across geographic regions and age groups. Young adults, particularly those aged 20-24 years, had the greatest absolute mortality burden and the largest absolute increase, whereas adults aged 50 years or older experienced the largest relative increases from substantially lower baseline rates. Men accounted for most deaths throughout the study period, although the year-by-sex interaction did not indicate a difference in the average log-linear slope.

This nationwide ecological analysis was designed to describe population-level motorcyclist mortality and its temporal distribution rather than individual rider risk or the causal effects of specific policies, exposures, or events. Earlier national studies captured the expansion phase of motorcycle-related mortality, including an approximately eightfold increase from 1996 to 2009 and an increase of more than 300% among motorcycle occupants from 2000 to 2008 [1-3]. Extending the national series through 2023 reveals a more complex trajectory, characterized by deceleration after 2010, decline after 2014, and renewed growth after 2020. The decline reported for tropical Latin America after the early 2010s is consistent with the middle portion of our series, but the subsequent Brazilian increase shows that this improvement was not sustained [4,5]. Thus, much of the apparent disagreement between previous reports of growth, stabilization, and decline likely reflects differences in the periods examined.

Geographic patterns further illustrate this changing trajectory. In 2023, age-standardized mortality was highest in the Northeast, Central-West, and North and lowest in the Southeast, consistent with previously identified high-burden clusters [4,6-9]. However, the Southeast showed the greatest average relative increase over the full study period despite having the lowest rate in 2023, reflecting its low baseline and steep early rise. Current burden and long-term relative change therefore describe different dimensions of the epidemic.

Sex and age patterns provide a similar distinction between absolute burden and relative change. Men accounted for 89.2% of deaths, and their 2023 mortality rate remained nearly eight times that of women. Although the male-to-female rate ratio decreased over time, the nonsignificant year-by-sex interaction refers only to the average log-linear slope and should not be interpreted as evidence that the nonlinear trajectories were equivalent. The segmented analyses showed differences in the timing and magnitude of individual temporal segments, while previous work has also documented increasing mortality among female motorcyclists in Brazil [10]. Age-related heterogeneity was more pronounced. Adults aged 20-24 years had the highest mortality rate in 2023 and the largest absolute increase, consistent with the established concentration of road-traffic mortality and years of life lost among young adults [3,17,18]. In contrast, adults aged 50 years or older had the largest relative increases from substantially lower baseline rates. Because these are age-specific rates, population aging alone does not explain this pattern. Changes in motorcycle exposure among older adults, cohort replacement, and greater vulnerability to severe injury are plausible contributors, but none was measured. The ICD-10 V20-V29 classification encompasses several motorized two-wheel vehicle types, including motorized bicycles [12], and the available mortality data cannot consistently distinguish conventional motorcycles from electrically powered two-wheel vehicle subtypes across the study period. The observed increase among older adults therefore cannot be attributed specifically to growth in e-bike use. Mortality also represents only the fatal component of the motorcycle injury burden. A Brazilian study documented a substantial increase in hospitalizations related to motorcycle crashes [19], reinforcing that the mortality trends observed here occur within a broader burden of nonfatal trauma. The present analysis did not assess nonfatal injuries, disability, or injury severity.

The temporal changes occurred against a changing road-safety and mobility context. Brazil strengthened drink-driving legislation through the 2008 “Lei Seca” [20], subsequently introduced the National Plan for the Reduction of Traffic Deaths and Injuries (PNATRANS) in 2018, and required helmet use for motorcycle riders and passengers throughout the study period. These measures provide relevant context but do not map uniquely onto the model-defined inflection points, and their individual effects cannot be estimated from this ecological analysis. Changes in motorcycle exposure are also relevant. Growth in the motorcycle fleet was associated with increasing mortality in the Federal District [21], and motorcycle registration has been associated with mortality across Latin American cities [22]. Brazilian surveillance studies have identified speeding, alcohol use, mobile-phone use, and traffic violations as potentially modifiable risks, while helmet legislation is associated with lower mortality and traumatic brain injury [23,24]. These external data identify plausible determinants but do not establish the mechanisms underlying the temporal or regional patterns observed here. More broadly, the Brazilian experience occurs within a substantial motorcyclist injury burden across Latin America and the Caribbean [5,22], although differences in motorcycle exposure, infrastructure, enforcement, vehicle composition, and mortality ascertainment limit direct cross-country comparisons.

The data-driven segmented model identified an inflection at 2020 followed by increasing mortality through 2023. Because 2020 was not prespecified as a breakpoint and only four subsequent annual observations were available, this finding should not be interpreted as evidence of an effect of the COVID-19 pandemic or any other specific event. Changes in mobility, employment-related motorcycle use, enforcement, and economic conditions during this period remain possible contextual explanations that require data specifically designed to evaluate them.

The principal interpretive limitation is the absence of direct measures of motorcycle exposure. Mortality rates were calculated using the resident population as the denominator rather than the number of motorcycle riders, registered motorcycles, trips, or vehicle-kilometres travelled. Consequently, the substantial increase in population-based mortality should not be interpreted as an equivalent increase in individual motorcyclist risk, and part of the temporal or regional variation may reflect changes in motorcycle use rather than exposure-adjusted mortality risk. The analysis also depended on the completeness and specificity of death certification and external-cause coding. Previous evaluations of the Brazilian Mortality Information System have documented limitations in completeness and cause-of-death specificity, and record-linkage studies have demonstrated underascertainment and reclassification of road-traffic deaths [25]. Discrepancies between official and corrected national mortality estimates have also been reported [26]. Misclassification of road-user type is particularly relevant because deaths assigned to V89 cannot be attributed to motorcycle occupants. The observed decline in V89 coding over time suggests improving vehicle-type ascertainment, but temporal and regional differences in nonspecific coding could still influence absolute rates and regional comparisons. The ecological design further precludes inference about individual-level risk factors.

Strengths of the study include nationwide coverage, 24 years of observation, a consistent case definition, age standardization to the 2022 census population, high completeness of age and sex information, and consistent findings across alternative models and sensitivity analyses. Together, these features provide a contemporary national description of motorcyclist mortality in Brazil while defining the limits within which the observed temporal, geographic, and demographic patterns should be interpreted.

## Conclusions

Motorcyclist mortality in Brazil reached its highest age-standardized rate in 2023 after a temporary decline from 2014 to 2020. These findings describe population-level mortality burden rather than exposure-adjusted risk per rider. The absolute burden remained concentrated among men and young adults: adults aged 20-24 years had the highest age-specific rate in 2023 and the largest absolute increase over the study period. Adults aged 50 years or older experienced larger relative annual increases, but from substantially lower baseline rates. The extent to which motorcycle exposure, road-user classification, and other temporally varying contextual factors contributed to these trends remains uncertain.

## Appendices

**Table 4:** Proportion of land-transport deaths assigned to an unspecified vehicle type (ICD-10 V89), by region and year, Brazil, 2000–2023 ICD-10, International Classification of Diseases, 10th Revision; V89, person injured in a transport accident involving an unspecified vehicle [12]. Values are percentages calculated as the number of deaths coded V89 divided by all land-transport deaths coded.

Region	2000	2001	2002	2003	2004	2005	2006	2007	2008	2009	2010	2011	2012	2013	2014	2015	2016	2017	2018	2019	2020	2021	2022	2023	% 2000-2023 (total)
North	20.7	19.9	23.6	24.8	26.6	23.8	26.3	26.8	27.4	28.5	25.8	25.0	21.5	20.9	24.2	19.4	24.8	14.5	18.2	23.6	25.7	27.0	23.9	19.6	23.3
Northeast	22.8	17.7	18.7	19.2	18.8	17.8	20.6	19.4	21.5	20.9	23.1	23.2	21.6	22.2	24.2	21.8	20.9	14.4	13.1	16.2	15.5	16.4	13.8	12.3	19.2
Southeast	47.8	41.9	39.6	36.4	33.9	33.7	21.0	22.4	21.0	18.9	19.6	18.8	20.9	19.5	19.8	17.7	21.7	17.6	17.8	16.3	17.1	16.5	18.6	16.2	23.9
South	37.0	30.1	31.6	28.4	25.5	24.4	22.7	21.5	21.0	21.2	22.3	19.9	17.9	16.8	16.0	14.4	10.4	7.8	5.3	4.2	6.0	10.4	8.5	4.0	18.1
Central-West	33.7	26.4	25.2	26.0	23.7	18.2	17.7	17.4	20.0	19.4	19.8	18.0	18.6	17.1	16.0	16.2	12.8	11.6	8.3	10.8	16.7	17.3	15.4	10.8	17.9
Brazil (Total)	36.8	31.5	30.7	29.2	27.3	25.9	21.3	21.3	21.5	20.6	21.6	20.6	20.4	19.7	20.5	18.5	18.9	14.1	13.2	14.3	15.5	16.5	15.6	12.7	20.9
